# Dyslipidemia and Associated Cardiovascular Risk Factors among Young Nepalese University Students

**DOI:** 10.7759/cureus.2089

**Published:** 2018-01-20

**Authors:** Gaurav Nepal, Eans T Tuladhar, Keshav Acharya, Aseem Bhattarai, Vijay K Sharma, Mithileshwor Raut, Binod K Yadav

**Affiliations:** 1 Maharajgunj Medical Campus, Tribhuvan University Institute of Medicine

**Keywords:** dyslipidemia, atherosclerosis, youth, nepal

## Abstract

Introduction

Cardiovascular diseases are one of the main causes of morbidity and mortality worldwide, atherosclerosis being the principal underlying cause of cardiovascular diseases. Early detection of dyslipidemia and long-term prevention of atherosclerosis by controlling risk factors should begin in young age. The purpose of this study was to assess dyslipidemia and associated cardiovascular risk factors among university students of Nepal.

Methods

A sample of 280 students aged 17–24 years, were selected randomly from Institute of Medicine, Tribhuvan University. An interview-based questionnaire was designed and information was collected on the basis of age, gender, smoking and alcohol consumption. Body mass index and waist-to-hip ratio of all participants were calculated. Fasting blood samples were collected from all participants and assayed for fasting serum total cholesterol, triglyceride, high-density lipoprotein and low-density lipoprotein.

Results

Overall, dyslipidemia was seen as hypercholesterolemia in 31 (11.1%), elevated low-density lipoprotein in 34 (12.1%), low high-density lipoprotein in 95 (33.9%) and hypertriglyceridemia in 39 (13.9%). Current smoking and binge drinking were significantly associated with hypercholesterolemia. Gender, binge drinking, and current smoking were found to be significantly associated with elevated low-density lipoprotein. All factors were significantly associated with hypertriglyceridemia. There was no statistically significant association between risk factors and the low high-density lipoprotein. Body mass index and waist-to-hip ratio were significantly higher in subjects with hypercholesterolemia, hypertriglyceridemia, and elevated low-density lipoprotein level.

Conclusions

The prevalence of dyslipidemia was high in young Nepalese university students. Screening the levels of lipids in youth, especially those at risk, and accurate follow-up of those with dyslipidemia can be done to reduce morbidity and mortality.

## Introduction

Cardiovascular diseases (CVDs) are one of the main causes of morbidity and mortality worldwide, atherosclerosis being the principal underlying cause of CVDs [1]. Dyslipidemia is one of the most important risk factors for atherosclerosis [2]. Elevated serum lipids lead to vessel wall responses, including endothelial dysfunction, smooth muscle cell proliferation, lipid accumulation, foam cell formation, and, eventually, necrosis and plaque development [3]. Although CVDs are not observed in childhood, cardiac risk factors such as dyslipidemia are present in children and they remain silent until adulthood [4]. Cardiovascular risk factors are on the rise in the Nepalese population and recent observation has shown a significant elevation of risk factors in the youth population [5].

Identification and prevention of cardiovascular risk factors at the young age is an important strategy to reduce present and future health risks. Creating a successful intervention that prevents or reduces dyslipidemia requires a better understanding of lipid pattern and associated cardiovascular risk factors. A population study of dyslipidemia among youth is lacking in our country. Information that will emerge from this study can fill this gap. Our aim in this study was to take an initiative towards determining the lipid pattern among young university students (17–24 years) in Nepal and to assess dyslipidemia and associated cardiovascular risk factors.

## Materials and methods

A cross-sectional study was carried out in Institute of Medicine (IOM), Tribhuvan University Teaching Hospital. The study was conducted from 20th February 2017 to 20th July 2017. The study population encompassed 280 undergraduate students aged 17–24 (156 males and 124 females). The study sample was statistically calculated based on 39% prevalence of dyslipidemia in a similar Egyptian study [7], 95% confidence interval, 5% acceptable margin of error. The estimated sample size was 380 but we could only achieve a sample of 280 as students were not willing to provide venous blood and it was difficult to maintain 12 hours overnight fasting. The sampling frame was undergraduate students from campuses affiliated to IOM and simple random sampling method was used to select the sample. Participants were selected if they were willing to participate, healthy, physically active and taking no medications known to influence lipid metabolism. The study was approved by the Institutional Review Board, IOM. Study objectives were explained and written informed consent was obtained from the participants prior to the study.

An interview-based questionnaire was designed by authors. For content validity, a pilot study was done among 10 volunteer medical students, three nursing students, and four faculty members who were subject experts, and who commented on the relevance and unambiguity of the questionnaire. After discussion of results and feedback, a final questionnaire was made. Information about age, gender, smoking and alcohol consumption was collected. Students who had smoked cigarette within the last 30 days were defined as current smokers. Similarly, binge drinking was defined as those who had five or more drinks on at least one occasion in the past month.

Height was measured by a non-stretchable plastic measuring tape after marking the subject to stand straight against an even wall. Body weight of all the subjects was measured by using a standardized weighing machine, which was calibrated in kilograms. Body mass index (BMI) was calculated as weight in kg/height in square meter. Waist circumference was measured using a non-stretchable plastic measuring tape over the unclothed abdomen, at the umbilicus level in the standing position. Hip circumference was measured over light clothing at the widest point over the buttocks when viewed from the side. Waist-to-hip ratio (WHR) was obtained by dividing the waist circumference by hip circumference.

Venous blood samples were obtained from the antecubital vein in suitable vacutainers at 08:00 am on the interview day after 12 hours overnight fasting. Triglycerides (TG), total cholesterol (TC) and high-density lipoprotein cholesterol (HDL) were analyzed enzymatically, using the kits provided by Human diagnostics and the low-density lipoprotein cholesterol (LDL) was calculated using Friedwald’s formula. All the estimations were done using the autoanalyser (BT 1500). Lipid profile of studied population was compared with the reference given by National Cholesterol Education Program, ATP III guidelines.

Data was collected in Microsoft Excel (Ver. 2013) and statistical analysis was performed using SPSS 21 (IBM Corp. Released 2012. IBM SPSS Statistics for Windows, Version 21.0. Armonk, NY: IBM Corp.). Chi-square-test and Fisher's exact test was used to identify differences between categorical variables. Multivariate Logistic regression analysis was used to examine the predictors of dyslipidemia. All results were described with odds ratios (ORs) and 95% confidence intervals (CIs). A p-value of less than 0.05 (two-sided) was considered to be statistically significant.

## Results

Overall, dyslipidemia was seen as hypercholesterolemia in 31 (11.1%), elevated LDL in 34 (12.1%), low HDL in 95 (33.9%) and hypertriglyceridemia in 39 (13.9%). In our study, 21 (7.5%) candidates were found to be current smokers and binge drinking was found in 87 (31.1%). The mean, standard deviation, and range of various lipid values in total samples are given in Table 1.

**Table 1:** Mean, standard deviation and range of serum lipids. TC: Total cholesterol; TG: Triglyceride; HDL: High-density lipoprotein cholesterol; LDL: Low-density lipoprotein cholesterol; SD: Standard deviation.

Lipids	Mean ± SD	Range
TC	3.68 ± 0.83	1.9–5.9
LDL	2.41 ± 0.72	0.9–​​​​​​​4.4
HDL	1.04 ± 0.23	0.2–​​​​​​​1.8
TG	1.14 ± 0.65	0.4–​​​​​​​5.3

Risk factors found to be significantly associated with hypercholesterolemia were a history of smoking (p < 0.01) and binge drinking (p < 0.001). Gender (<0.05), binge drinking (<0.001), and smoking (<0.001) were found to be significantly associated with elevated LDL. There was no statistically significant association between risk factors and the low HDL. All factors (<0.05) were significantly associated with hypertriglyceridemia. Details of the bivariate analyses of the various factors with dyslipidemia are shown in Tables 2-5.

**Table 2:** Risk factors associated with hypercholesterolemia. OR: Odds ratio; CI: Confidence interval.

Factors	Sample size	Hypercholesterolemia	p-value	OR	95% CI
Overall	280	31 (11.1%)			
Gender					
Male	156	22 (14%)	>0.05	0.48	0.21– 1
Female	124	9 (7.3%)			
Binge drinking					
Yes	87	18 (20.7%)	<0.01	3.6	1.7– 7.8
No	193	13 (6.7%)			
Current smoking					
Yes	21	9 (42.8%)	<0.001	8	3–21.3
No	259	22 (8%)			

**Table 3:** Risk factors associated with elevated LDL. LDL: Low density lipoprotein cholesterol; OR: Odds ratio; CI: Confidence interval.

Factors	Sample size	Elevated LDL	p-value	OR	95% CI
Overall	280	34 (12.1%)			
Gender					
Male	156	25 (16%)	<0.05	0.41	0.12– 1
Female	124	9 (7.3%)			
Binge drinking					
Yes	87	22 (25.3%)	<0.001	5.1	2.4– 11
No	193	12 (6.2%)			
Current smoking					
Yes	21	11 (52%)	<0.001	11.3	4.3–30
No	259	23 (9%)			

**Table 4:** Risk factors associated with low HDL. HDL: High density lipoprotein cholesterol; OR: Odds ratio; CI: Confidence interval.

Factors	Sample size	Low HDL	p-value	OR	95% CI
Overall	280	95 (33.9%)			
Gender					
Male	156	48 (30.8%)	>0.05	0.73	0.45– 1.2
Female	124	47 (37.9%)			
Binge drinking					
Yes	87	23 (26.5%)	>0.05	1.65	0.95–2.9
No	193	72 (37.3%)			
Current smoking					
Yes	21	6 (28.5%)	>0.05	1.3	0.5–​​​​​​​3.5
No	259	89 (34.5%)			

 

**Table 5:** Risk factors associated with hypertriglyceridemia. OR: Odds ratio; CI: Confidence interval.

Factors	Sample size	Hypertriglyceridemia	p-value	OR	95% CI
Overall	280				
Gender					
Male	156	28 (17.9%)	<0.05	0.45	0.21–0.95
Female	124	11 (8.9%)			
Binge drinking					
Yes	87	16 (18.4%)	<0.05	1.67	0.83–​​​​​​​3.4
No	193	23 (11.9%)			
Current smoking					
Yes	21	7 (33%)	<0.05	3.5	1.34–​​​​​​​9.5
No	259	32 (12%)			

Multivariate logistic regression analyses of the associations between risk factors and dyslipidemia are shown in Table 6 including the ORs and CIs of the different variables. Risk factors were subjected to the logistic-regression model, using the ‘‘Enter’’ method. Multivariate analysis showed that current smoking and binge drinking were significant predictors of hypercholesterolemia and elevated LDL.

**Table 6:** Predictors of dyslipidaemia by multivariate logistic regression analysis. LDL: Low density lipoprotein cholesterol; HDL: High density lipoprotein cholesterol; OR: Odds ratio; CI: Confidence interval. Values in bold: p < 0.05

	Hypercholesterolemia		Elevated LDL		Hypertriglyceridemia		Low HDL	
Factors	OR	95% CI	OR	95% CI	OR	95% CI	OR	95% CI
Gender								
Male	1	0.37–2.65	1	0.4–​​​​​​​2.8	0.5	0.23–​​​​​​​1.2	0.85	0.5–​​​​​​​1.5
Female								
Binge drinking								
Yes	2.25	0.85–​​​​​​​6	3.2	1.2–​​​​​​​8.2	0.9	0.4–​​​​​​​2.2	1.5	0.83
No								
Current smoking								
Yes	4.85	1.6–​​​​​​​14.6	5.8	2–​​​​​​​17	2.8	0.9–​​​​​​​8.6	0.87	0.3–​​​​​​​2.5
No								

Pearson's correlation analysis showed that in females, only the serum LDL level was significantly correlated with BMI (r = 0.2, p < 0.05), however, serum TC and LDL levels both significantly correlated with WHR (r = 0.265, r = 0.294, p < 0.001). Meanwhile, in the case of males, all TC, TG, LDL, HDL levels significantly correlated with BMI (r = 0.239, r = 0.217, r = 0.186, r = 0.164, p < 0.001) and WHR (r = 0.416, r = 0.175, r = 0.348, r = 0.332, p < 0.001).

## Discussion

Dyslipidemia is one of the important risk factors for CVD. Assessment of the prevalence of dyslipidemia in youth population is an important step towards designing a primary prevention program for CVD. This is one of the first studies to the assess dyslipidemia among young Nepalese university students.

The overall prevalence of hypercholesterolemia, elevated serum LDL, low serum HDL, and hypertriglyceridemia in this study was 31 (11.1%), 34 (12.1%), 95 (33.9%), and 39 (13.9%), respectively. A similar study done in Kingdom of Saudi Arabia showed that the prevalence of hypercholesterolemia, hypertriglyceridemia, high LDL, low HDL among university students (17–26 years ) was 17.7%, 5.0%, 16.8%, and 46.3%, respectively [6]. Our current study rates are lower than this study, except for hypertriglyceridemia. A study among university students (17–27 years) in Egypt, reported that the hypercholesterolemia prevalence was 38.8%, hypertriglyceridemia 29.7%, low HDL-C 27.1%, and high serum LDL 33.1% [7]. The current study rates are lower than Egyptian study, except for low serum HDL. Our figures are lower: this may be due to the young age group of our study participants (17–24 years) and modernization and accumulation of risk factors and quality of life of university students of Egypt and Saudi Arabia.

Elevated serum LDL and hypertriglyceridemia were significantly associated with gender. Sexual dimorphism in lipid metabolism seems to be the result of a complex network of hormone action in combination with other direct or indirect modulators of lipid metabolism like insulin and adipokines, gene expression and imprinting, etc. that need to be explored in the future [8-9].

The current study revealed a significant association of hypercholesterolemia, high serum LDL, and hypertriglyceridemia with cigarette smoking. The rise in lipid levels in smokers may be explained by the following mechanism: cigarette smoking causes absorption of nicotine into the body which leads to lipolysis and release of free fatty acids into the blood stream via nicotine stimulated secretion of catecholamines. These increased free fatty acids in liver give rise to increased hepatic triglyceride and very low-density lipoprotein cholesterol (VLDL) synthesis, thus increasing the concentration of lipids in blood [10-11]. Smoking increases insulin resistance and thus, causes hyperinsulinemia. LDL, VLDL, and triglyceride are elevated in hyperinsulinemic conditions due to decreased activity of lipoprotein lipase [12].

In the present study, all forms of dyslipidemia except low HDL were significantly associated with binge drinking. Oxidative stress caused by excess ethanol consumption promotes pro-inflammatory cytokines in the Kupffer cells and adipocytes. These cytokines promote inflammation, insulin resistance, and dyslipidemia [13]. Excess ethanol consumption is also associated with inhibition of adenosine monophosphate-activated protein kinase (AMPK). It is a master regulator of metabolism that senses oxidative stress. Activation of AMPK increases fatty acid oxidation and inhibits fatty acid synthesis, whereas its inhibition blocks fatty acid oxidation and promotes fatty acid synthesis [14-15].

In the current study, a significant correlation of anthropometric parameters with lipid parameters was seen. It is documented in previous studies that obesity is a major cause of dyslipidemia [16-18]. Obesity-associated dyslipidemia is atherogenic as obese individuals have increased atherogenic small, dense LDL particles and elevated levels of apolipoprotein B [19]. It has been proved from epidemiological studies that the association between dyslipidemia and abdominal obesity is mediated through an etiopathological mechanism [20]. So, BMI and WHR may be considered screening tools to detect dyslipidemia in individuals at the early stage. By logistic regression analysis, we found that current smoking and binge drinking were significant predictors of hypercholesterolemia and elevated serum LDL in the young population. Fortunately, both of these risk factors are preventable.

It has been shown that prevalence of risk factors for non-communicable diseases in childhood and adolescence increases the tendency towards development of disease in adulthood [21]. Thus, screening adolescents and young adults for dyslipidemia especially high risk overweight and obese ones is of paramount importance. A study has reported that non-optimal levels of high LDL and low HDL cholesterol during young adulthood are independently associated with CVD two decades later [22].

The main strength of the present study includes confirmation of high dyslipidemia prevalence among the young Nepalese university students, making it necessary to develop effective preventive measures. We recommend intervention programmes to university students even at an earlier age through screening, health education, and counseling. Increasing student awareness of the importance of adopting healthy dietary habits and increasing physical activity is of paramount importance to reduce dyslipidemia prevalence and prevent atherosclerosis/CVD.

This study has its own limitations. The major one being that we did not assess social and cultural influence, genetic influence/familial history, the influence of some factors such as nutrition, physical activity, and diet on dyslipidemia. The sample size of our study was relatively small so we may not generalize the result for the whole country. Also, apoB and apoA-1 levels were not measured. However, most, but not all, studies point out that measurement of apoB and apoA-1 for universal screening provides no additional advantages over measuring LDL and HDL [2].

## Conclusions

The prevalence of dyslipidemia was high in young Nepalese university students (17–24 years). Dyslipidemia was seen as hypercholesterolemia in 31 (11.1%), elevated LDL in 34 (12.1%), low HDL in 95 (33.9%), and hypertriglyceridemia in 39 (13.9%). Early detection of dyslipidemia by screening and long-term prevention of cardiovascular disease by controlling the risk factors should begin in youth. Our study also found that 21 (7.5%) candidates were current smokers and 87 (31.1%) were involved in binge drinking. Increasing student awareness of the importance of controlling alcohol consumption, quitting smoking, and increasing physical activity is of paramount importance to reduce dyslipidemia prevalence and prevent cardiovascular disease.
